# Pupil’s perspectives on female genital cutting abandonment in Harari and Somali regions of Ethiopia

**DOI:** 10.1186/s12905-018-0653-6

**Published:** 2018-10-17

**Authors:** Asresash D Abathun, Johanne Sundby, Abdi A Gele

**Affiliations:** 1 Faculty of Medicine, Institute of Health and Society, University of Oslo, Post-box 1130 Blindern, 0318 Oslo, Norway; 2 Department of community Medicine and global Health, Institute of Health and Society, University of Oslo, PO Box 1130 Blindern, 0318 Oslo, Norway; 3 Department of Nursing and Health Promotion, Oslo and Akershus University College, Oslo, Norway; 4 Norwegian Center For Minority Health Research, Oslo University HospitalVisiting, Aker sykehus, Trondheimsveien 235, 0586 Oslo, Bygg 6 (8. etasje) Norway; 5 NAKMI, Oslo universitetssykehus, Aker, Postboks 4959 Nydalen, 0424 Oslo, Norway

**Keywords:** Attitude, Abandonment, FGC, Government, Family, Education

## Abstract

**Background:**

Female Genital Cutting (FGC) is a harmful traditional practice that affects the physical and mental health of girls and women in many ways. In Ethiopia, although both governmental institutions and None-Governmental- Institutions (NGOs) launched different campaigns against FGC, their effects on the peoples’ attitudes towards the practice have not been deeply investigated yet. Hence, this study particularly aimed to investigate the pupils’ perspectives on FGC abandonment in the Harari and the Somali Regional States of Ethiopia where the prevalence of the practice was thought to be high.

**Methods:**

A school-based cross-sectional study was conducted in the Somali and the Harari Regional States of eastern Ethiopia from October to December 2015. While purposive sampling was implemented to select the study areas from the two Regional States, stratified random sampling method was used to select 480 study subjects from those areas.

**Results:**

The findings showed that the participants who received information through multiple information channels were more likely to support the abandonment of FGC than those who received information from a single source (*p* < 0.05). Similarly, the findings indicated that school-based awareness campaigns and TV-based media communications were the main sources of information that influenced a high proportion of young people to support the abandonment of the practice. The findings revealed that the majority of the participants strongly supported the abandonment of FGC.

**Conclusions:**

Multiple information channels that include school-based awareness campaigns were found to be the best way to support the abandonment of FGC. Although the study shows an impressive improvement among the school girls and boys in recognizing the harmful effects FGC, complete abandonment of the practice might not be easily achieved due to its deep-rooted nature. Thus, to quicken the perpetuation of FGC in the stated Regional States, awareness creating campaigns that change the attitudes of youths towards the practice should be delivered through various sources. In this regard, school-based education, school mini-media, social media, and using the co-curricular activities to uncover the danger of this harmful practice could play significant roles in changing the pupils’ attitudes towards the practice.

**Electronic supplementary material:**

The online version of this article (10.1186/s12905-018-0653-6) contains supplementary material, which is available to authorized users.

## Background

Female Genital cutting (FGC) is a harmful traditional practice which affects the physical and mental health of girls and women [1]. FGC affects girls’ schooling and limits their capacity [2]. It is an issue in both the development and governance, and therefore ending all forms of FGC is the interest of families, communities, and nations [3].

It is estimated that more than 200 million girls and women alive today have undergone female genital cutting in the countries where the practice is concentrated [1]. Furthermore, there are an estimated 3 million girls at risk of undergoing female genital mutilation/cutting every year. FGC has been documented in 30 countries, mainly in Africa, as well as areas in the Middle East and Asia [1]. Some forms of FGC have also been reported in certain ethnic groups in South America [1]. Moreover, FGC has been practiced by migrants living in Europe, Australia, and North America [1]. Even if the worldwide decline in FGC is maintained at current rates, population growth means that about 196 million girls would be cut by 2050 [4].

World Health Organization (WHO) classified FGC into four groups. Type I,Sunna/*clitoridectomy*, which is the partial or total removal of the clitoris and/or the prepuce. Type II or excision involves the partial or total removal of the clitoris and the labia minora with or without excision of the labia majora. Type III or infibulation involves in narrowing of the vaginal opening through the creation of a covering seal. Type IV includes all other harmful procedures to the female genitalia for non-medical purposes [5, 6]. Some studies revealed that there was a low level of awareness towards the types of FGC practice, and there had been an opinion that some types are not harmful [7, 8].

Although the numbers of girls and women affected by FGC, is distressing, a recent report by United Nation Children’s Fund (UNICEF), indicates that there has been a slow decline in the overall prevalence of FGC over the past decades. Moreover, the same report shows that younger women, in 28 African countries, are less likely than older women to have experienced any form of FGC, indicating a generational shift towards ending the practice [9]. Despite the progress made in some countries, many girls are still at risk of FGC, due to its association with marriageability, religion, chastity, female honor, beauty, and aesthetics.

### Factors that influence the abandonment of FGC

Although women are the victims of the procedure, they also have the traditional obligation to perform the procedure on their daughters. One of the most common explanations for the continuation of FGC is the perception that FGC is a local custom, and thus it should be maintained. Women are often saying that they are unwilling to change these customs since they have always done it, and have grown up this way [10]. In communities where FGC is a criterion for marriage, nearly all girls are circumcised. Families allow the procedure (FGC) on their daughter, in order to prepare them for adulthood and to ensure to have a proper marriage. It is also important in light of the economic benefit, and to be respected by the community. On the other hand, uncircumcised girls are often stigmatized and sometimes divorced. Under this condition, FGC can be seen as the best choice for the parents to ensure that their daughter can have a happy future. The girls in these communities also wish to be circumcised, thinking that they are better off with the FGC [11]. In such a society where the social obligation to cut their daughter is in place, expecting a negative attitude toward FGC cannot be possible in changing the behavior of the individual or one family. Another study also suggested that social pressure and fear of rejection from the community were found to be significant barriers to the abandonment process [11].

The research identified that education has a strong impact in changing attitudes and influencing a family’s choice to have FGC performed [12]. In addition to education, legislative developments, including new constitutions and other emerging mechanisms are critical to FGC abandonment. Because of many cases of FGC and the risks to health and life, FGC is now outlawed in most of the global nations [4, 10].

Communication that effectively merges media and community interventions will result in changing behavior or attitudes towards FGC [13]. A study in Egypt revealed that communities who have had access to information about FGC through the printed media, Television, community meeting, and in the mosque/church gatherings have more likelihood of supporting the discontinuation of FGC when contrasted with peers lacking access to information about FGM/C [14].

Ethiopia is one of the countries in which the United Nation Population Fund (UNFPA) and UNICEF Joint Programme for the Accelerated Abandonment of Female Genital Cutting (FGC) are being implemented. The program brings expertise in the area of the social norm to support the process of positive social transformation which includes empowering, education, community dialogue, and public commitments to abandon the practice [15]. The government of Ethiopia recognized that the abandonment of FGC requires a human right based approach and a coordinated joint action. In 2014, the Government of Ethiopia committed to ending FGC by 2025 and has been working on eliminating the practice through public information campaigns [16]. Although a lot has been done in Ethiopia, the challenges of eradicating FGC is apparent, mainly in tackling the many and complicated cultural beliefs by the society [17]. Despite the effort made by the Ethiopian government in ending FGC, there is no research about what works and what does not towards the abandonment of FGC, in Somali and Harari regions of eastern Ethiopia, where the prevalence is highly pronounced. Therefore, this study aims to investigate pupil’s perspectives toward the abandonment of FGC, particularly the source of information that impact pupils’ attitude toward the practice in Somali and Harari regions, eastern Ethiopia.

## Methods

### Study design

A school-based cross-sectional quantitative study was carried out in Jigjiga town of the Somali region and the Harar town of the Harari Region from October to December 2015.

### Setting

Jigjiga town from Somali region and Harar town from Harari region were purposely selected, by considering that both areas are the capital cities, and accessible to the information sources such as media and internet which might help to get useful information. Jigjiga town is the capital city of the Somali region and located approximately 635 *km* away from Addis Ababa. It has two public and three private high schools and ten primary schools. The Harar town, which is 530 *km* away from Addis Ababa, is the capital city of Harari Regional State. There are four public and two private high schools and more than 15 primary schools in the town. One secondary school and one primary school were randomly selected from all available primary and secondary schools, due to budget constraints and unfortunately, all selected schools were public schools.

### Study population

Sampled Girls and Boys who are attending primary and secondary schools from the selected schools.

### Sample

The sample size is calculated using the formula for two population proportions. To achieve a 95% confidence level with 80% statistical power, and to detect a 5% difference in the proportions, the calculation yields to a sample of 480 with 20% non-response rate. We stratified the study subjects into gender strata, to ensure that an equal number of units are selected from girls and boys.

### Data collection

Ten nurses (five males and five females), were selected for the data collection, and two supervisors were assigned to oversee the data collection process, in addition to the principal investigator. Questionnaires were developed in English and validated through several rounds with the advisors, and experts with prior experience in FGC study in Ethiopia. In order to make respondents understand the questions well, questionnaires were translated into native languages of participants, in this case, Amharic, and Somali, and back-translated into English, to assure the consistency and the correctness of the translation. While Somali translated version was used in the Somali region, the Amharic version was used in Harari region. The questionnaires included both socio-demographic details, and questions that assessed factors such as participants’ knowledge, attitude, and practice, types of FGC, Reason and information sources. The dependent variable in this study is support or/not support the abandonment of FGC.

Training was given to data collectors, on how to collect data, prior to data collection. For further validation of the questionnaire, a pre-test was administered to 80 students (boys and girls) in different schools, which were not included in the study. In addition to this, it was tried to strengthen the validity of the study, by recruiting field assistance, and data collectors from the local community. Inclusion criteria for the study were being male and female, enrolled in primary and secondary schools, with the age range of 16 to 22 years, and willing to participate in the study. In some setting 16 years might not be feasible for primary schools, but in Ethiopia, this setting is common due to repetition or temporary dropout. In addition, the reported age data may be erroneous due to the lack of birth certificates.

### Data analysis

Once collected, the data were computerized and analyzed in the Statistical package for social science (SPSS) Version 20. We used cross-tabulations, to determine group differences. Afterward, the Chi-square test was performed to determine whether the variables are statistically independent or if they are associated. Variables that were statistically significant (*p* < 0.05), in the bivariate analysis, were fitted in the final multivariable logistic regression model. Crude and adjusted odds ratios with 95% CI were calculated to measure the strength of association between the dependent and independent variables.

## Results

### Socio-demographic characteristics

Four hundred and eighty questionnaires were distributed by hand, and 478 were returned giving a response rate f 99.6%. The majority of the respondents were Females 246(51.3%) and secondary education respondents 250(52.3%). The mean age of study participants was 17.4 (SD 1.34) (Table 1).

**Table 1:** Socio-demographic characteristics of the respondents in Somali and Harari regions, Eastern Ethiopia

Characteristics	Frequency (*N* = 478)	Percent (%)
Age		
16–18	388	81.2
19–20	80	16.7
21–22	10	2,1
Gender		
Male	232	48,7
Female	246	51.3
Region		
Somali	238	49.8
Harari	240	50,2
School		
Primary	228	47.7
Secondery	250	52.3

### Source of information about FGC

The majority of the respondents noted that they received information about FGC from different sources. These include family members 288(63.3%), schools 255(53.3), through the television 208(43.5%), friends 187(39.1%), the internet 141(29.5%), and newspapers 106(22.2%). In the inter-region analysis, about 163(69%) of the Somali respondents got information from the family, while in the Harari region, almost 102(43%) of them got information from the internet. In both regions, school-based education 255(53.3%) is found to be the highest source of information. The inter-sex analysis showed 167(67.9%) of female respondents got information from family members (Table 2).

**Table 2:** Source of information of respondents in Somali and Harari regions, eastern Ethiopia

Source of information about FGC	Total Frequency *N* = 478	Percent(%)	Regions		Gender	
			Harari region *N* = 240	Somali region *N* = 238	Male *N* = 232	Female *N* = 246
From Family members	288	63.3	125 (52.1%)	163 (68.5%)	121 (52.2%)	167 (67.9%)
From Friends	187	39.1	76 (31.7%)	111 (46.6%)	82 (35.3%)	105 (42.7%)
In the classroom	255	53.3	118 (49.2%)	137 (57.6%)	120 (51.7%)	135 (54.9%)
From Health professionals	147	30.8	77 (32.1%)	70 (29.4%)	87 (37.5%)	60 (24.4%)
rom Bulletin board	122	25.5	73 (30.4%)	49 (20.6%)	70 (30.2%)	52 (21.1%)
From Internet	141	29.5	102 (42.9%)	39 (16.4%)	76 (32.8%)	65 (26.4%)
From Health education	151	31.6	66 (27.5%)	85 (35.7%)	83 (35.8%)	68 (27.6%)
From Television	208	43.5	100 (41.7%)	108 (45.4%)	106 (45.7%)	102 (41.5%)
From News paper	106	22.2	67 (27.9%)	39 (16.4%)	62 (26.7%)	44 (17.9%)
From Radio	163	34.1	61 (25.4%)	102 (42.9%)	88 (37.9%)	75 (30.5%)

All percentage in the source of information are above 100 due to Multiple response

### Circumcision status of study participants and their knowledge about FGC

The majority of the respondents 219(45.8%) reported that 6 to 14 years were the age of circumcision in their regions. However, 114(23.8%) of the respondents claimed that they didn’t know the age at which circumcision was done. About 26(11%) of the respondents from the Harari region mentioned that the Sunna type of FGC was performed in their region. Similarly, 86(36.1%) of respondents from the Somali region reported that type III FGC (infibulations) was the major type of FGC in their region. However, in both regions, 218(45.6%) of the respondent claimed that they had no knowledge of the types of FGC performed in their regions, and the majority 172(71.7%) of them were from the Harari region. About 351(73%) of the respondents reported traditional practitioners were the major performer of FGC in their regions, while only 17(3.6%) reported health professionals. Mothers 197(41.2%) were found to be the major decision makers of whether or not FGC is performed in both regions (Additional file 1).

About 79(32.1%) of girls were circumcised from both regions, of whom 58(51.3%) of them were from the Somali region while 21(15.8%) were from the Harari region. The majority of the circumcised girls 53(67.1%) reported that the decision was made by the mother and 43(54.4%) of them responded that the circumcision helped them, of whom 37(63.8%) were from the Somali region (Table 3).

**Table 3:** FGC status of female respondents in Somali and Harari regions, eastern Ethiopia

Indicators	Total *N* = 246	percentage	Regions	
			Harari	Somali
Are you circumcised				
Yes	79	32.1	21 (15.8%)	58 (51.3%)
No	167	67.9	112 (84.2%)	55 (48.7%)
If Yes who made the decision(*N* = 79)				
Father	7	8.9	4 (19.0%)	3 (5.2%)
Mother	53	67.1	14 (66.7%)	39 (67.2%)
Grand mother	15	19	2 (9.5%)	13 (22.4%)
I my self	4	5.1	1 (4.8%)	3 (5.2%)
Does FGC helped you				
Yes	43	54.4	6 (28.6%)	37 (63.8%)
NO	18	22.8	6 (28.6%)	12 (20.7%)
I don’t know	18	22.8	9 (42.9%)	9 (15.5%)

### Support towards the abandonment of FGC

Three hundred ninety-one (82%) of the study sample supported the abandonment of FGC, and 381(97.4%) reported that community awareness is the best strategy to abandon the practice. Two hundred one (42%) of young people teach the community about the harmful effect of FGC, and 73(15.3%) of them exposed the performers to the police. The study participants mentioned that the family 273(57.5%) was the responsible body in abandoning FGC; followed by the government 246(51.8%) (Additional file 1).

### Group differences in support toward the abandonment of FGC

A chi-square test was performed to assess the relationship between the independent variables with the support or/not support the abandonment of FGC (see Table 4). Based on the result, statistically significant differences were observed in the support toward the abandonment of FGC among, participants who heard FGC information from different sources 292(84.1%) versus with those who heard information from one source 99(75.6%), *X*^*2*^*(1) = 4.699*, *p* = 0.03, participants with secondary level education 218(87.2%) versus with primary level education 173(75.9%), *X*^*2*^*(1) = 10.269*, *p* = 0.001, participants with higher age group [15–20] 273(87.2%) versus with the lower age group (16 years) 118(71.5%), *X*^*2*^*(1) = 17.900*, *p* < 0.001, participant with Somali ethnic group 185(86.9%) versus with other ethnic group 159(76.4%), *X*^*2*^*(2) = 7.682*, *p* = 0.021, participants with no knowledge of the types of FGC 164(75.2%) versus with those with the knowledge of the types of FGC, X^2^(2) = 13.076, *p* = 0.001 and participants who reported the major reason to perform FGC as a tradition 323(81.8) versus with those who mentioned other reasons, *X*^*2*^*(2) = 9.298*, *p* = 0.010. The result also showed that there was no statistically significant difference observed in the support toward the abandonment of FGC among boys and girls, *X*^*2*^*(1) = 1.536*, *p* = 0.215, in the religion of the participants, *X*^*2*^*(2) = 3.520*, *p* = 0.172 and in the circumcision status of the girls, *X*^*2*^*(1 = 246) = 1.076*, *p* = 0.300 (Table 4).

**Table 4:** Group differences in support toward abandonment of FGC in Somali and Harari regions,eastern Ethiopia

Variables (*n* = 478	Support to abandon FGC	Do not support the abandonment	X^2^	*P*-value
Gender of participants				
Male	195(84.1%)	37(15.9%)	1.536	0.215
Female	196(79.7%)	50(20.3%)		
Information heard about FGM/C				
Heard from one source	99(75.6%)	32(24.4%)		
Heard from different sources	292(84.1%)	55(15.9%)	4.699	0.030*
Educational status				
Primary level education	173(75.9%)	55(24.1%)		
Secondary level education	218(87.2%)	32(12.8%)	10.269	0.001*
Age of participants				
16 years	118(71.5%)	47(28.5%)		
17–22 years	273(87.2%)	40(12.8%)	17.900	0.001*
Ethnicity of participants				
Somali	185(86.9%)	28(13.1%)	7.682	0.021*
Harari	47(82.5%)	10(17.5%)		
Others	159(76.4%)	49(23.6%)		
Type of FGC performed in the region				
Sunna	119(90.2%)	13(9.8%)		
Infibulations	108(84.4%)	20(15.6%)		
I don’t know	164(75.2%)	54(24.8%)	13.076	0.001*
Reason to perform FGC				
Tradition	323(81.8)	72(18.2%)	9.298	0.010*
Religious requirmrnt	47(92.2%)	4 (7.8%)		
I don’t know	21(65.6%)	11(34.4%)		
Religion				
Muslim	260(84.1%)	49(15.9%)	3.520	0.172
Orthodox	117 (77%)	35 (23%)		
Others	14 (82.4%)	3 (17.6%)		
Circumcision status of girls (*n* = 246)				
Circumcised	66 (83.5%)	13 (16.5%)	1.076	0.300
Not circumcised	130 (77.8%)	37 (22.2%)		

*Statistically significant at *p* < 0.05)

Bivariate and multivariable logistic regression analyses were done to identify independent variables that show significant association in the support toward the abandonment of FGC. All variables which showed statistically significant association with *p*-value < 0.05 during the bivariate analysis were entered to multivariable logistic regression analysis and significance was decided at *p*-value < 0.05 (Table 5).

**Table 5:** A logistic regression analysis for the support of FGC abandonment with different categorical variables in Somali and Harari regions, eastern Ethiopia

Variables (*n* = 478)	Support to abandon FGC	Do not support the abandonment	Crude OR	*P*-value	Adjusted OR with CI	*P*-value
Source of formation heard about FGM/C						
Heard from one source	99(75.6%)	32(24.4%)	0.583 (0.356–0.953)	0.031	0.567 (0.333–0.967)	0.037*
Heard from different sources	292(84.1%)	55(15.9%)	1		1	
Educational status						
Primary level education	173(75.9%)	55(24.1%)	0.462 (0.286–0.746)	0.002	0.506 (0.292–0.879)	0.016*
Secondary level education	218(87.2%)	32(12.8%)	1		1	
Age of participants						
16 years	118(71.5%)	47(28.5%)	0.368 (0.229.591)	0.001	0.536 (0.303–0.949)	0.032*
17–22 years	273(87.2%)	40(12.8%	1		1	
Type of FGC performed in the region						
Sunna	119(90.2%)	13(9.8%)	3.014 (1.574–5.773)	0.001	2.574 (1.186–5.587)	0.017*
Infibulations	108(84.4%)	20(15.6%)	1.778 (1.008–3.137)	0.047	1.347 (0.701–2.590)	0.371
I don’t know	164(75.2%)	54(24.8%)	1		1	
Ethinicity						
Somali	185(86.9%)	28(13.1%)	1		1	
Harari	47(82.5%)	10(17.5%)	0.711 (0.323–1.567)	0.398	1.142 (0.472–2.763)	0.769
Others	159(76,4%)	49(23.6%)	0.491 (0.295–818)	0.006	1.007 (0.523–1.941)	0.983
Reason to perform FGC						
Tradition	323(81.8%)	72(18.2%)	1			
Religious requirement	47(92.2%)	4 (7.8%)	2.619 (0.914–7.)502	0.073	2.129 (0.715–6.334	0.174
Others	21(65.6%)	(34.4%)	0.426 (0.196–0.922)	0.30	0.673 (0.291–1.555)	0.354

*Statistically significant at *p* < 0.05)

As indicated in Table 5, In the multivariable logistic regression analysis, Information sources, educational status of the participants, the age of the participants, and knowledge of the type of FGC had statistically significant association (*p*-value < 0.05) with the support toward the abandonment of FGC. Accordingly, participants who got information only from one source (AOR = 0.567, 95% CI = 0. 333–0.967) were less likely to support the abandonment of FGC when compared to those who got information from multiple sources. Similarly, Participants with primary level education (AOR = 0.506, 95% CI = 0.292–0.879) were less likely to support the abandonment of FGC when compared to those with secondary level of education. Participants in the lower age group (16 years) (AOR = 0.536, 95% CI = 0. 303–0. 949) were also less likely to support the abandonment of FGC than participants in the higher age group (17–22 years). Furthermore, those participants with the knowledge of the Sunna type of FGC (AOR = 2.574 95% CI =1.186–5. 587) were nearly three times more likely to support the abandonment of FGC than others. While the ethnicity of the participants (AOR = 1.007 95% CI = 0.523–1.941), and reason to perform FGC (AOR = 2.129 95% CI = 0.715–6.334) were not significantly associated (*p*-value > 0.05) with the support toward the abandonment of FGC in the multivariable logistic regression analysis (Table 5).

## Discussions

This study examined pupil’s perspectives toward the abandonment of FGC in Somali and Harari regions of eastern Ethiopia. The findings of this study provide important information, on the opportunities for intervention and to formulate efficient FGC programs in regions with high prevalence of FGC in Ethiopia. According to the study result, participants who got information only from one source were less likely to support the abandonment of FGC when compared to those who have received information from different sources (AOR = 0.567, 95% CI = 0.333–0.967). This implies that the more people are exposed to different sources of information, the higher the probability of knowing the harmful effect of FGC, and change their attitudes toward the practice. This study is supported by a study on FGC in the United States, Ghana, Gambia, and Kenya, which stated that mass media were the main source of information which influences the process of understanding about the practice among the general public as well as policymakers [18]. Other studies have also supported our findings by stating that people who are aware of the negative health consequences of FGC are more likely to support the discontinuation of FGC [14, 18, 19].

Surprisingly, this study shows that most of the girls reported that they were not circumcised, and the majority of them were from the Harari region. While 15.8% of girls at Harar were circumcised, over half of the girls in the Somali region, have experienced FGC. The high number of circumcisions in the Somali region might be due to the deep-rooted culture, whereby the family is the decision maker for circumcisions, and the practice is considered as the criteria for marriage. This finding was supported by the qualitative study done in Somali and Harari regions, where the majority of women discussants noted that they perform the procedure due to fear of the stigma and discrimination by the community, and to prepare their daughter for a proper marriage [20]. Another study also supported by stating that the family is the one who decided for circumcision. Which was seen as the cut status of a mother is associated with the cut status of her daughter [21].

The high number of uncircumcised girls may be due to the awareness creation at the school level, at the community level and the information which they (the girls) got from multiple sources may help them to recognize the harmful effect of FGC. This study is supported by other studies which stated that the prevalence of FGC among girls is declining and the decision to send girls to school (for education) plays a great role in changing the positive attitude of girls toward FGC [22–24]. UNICEF reported that FGC prevalence rates among girls aged 15 to 19 have declined in some African countries, and FGC should end. There has been an overall decline in the prevalence of the practice over the last three decades among girls and women, but not all countries have made progress, and the pace of decline has been uneven [25, 26]. These indicated that there must be the subsequent legislation against FGC, and providing convincing reasons to abandon the practice, by strengthening a partnership among governments, religious leaders, an influential people, young people, and the schools, to transfer knowledge about the harmful effects of FGC to end the practice.

The present study also shows that Participants with primary level education and those with lower age group (16 years) were less likely to support the abandonment of FGC when compared to those with secondary level education and with a higher age group (17–22 years) respectively. This indicated that children in the lower age are more influenced by their parents than their peers. Decision-making processes for FGC are often led by older female relatives, including mothers and grandmothers, as they have the sole responsibility of making this happen as a sign of fulfilling their social responsibility. In the Ethiopian context, where there is respect for families, it is extremely difficult for younger children to influence those decisions. This study is supported by WHO fact sheet, which stated that FGM/C is mainly carried out on young girls under the age of 15 years old who often have little choice in the matter [27]. Another study also stated that older mothers had more positive attitudes toward female genital mutilation, felt more social pressure and had a greater intention to allow their daughters to undergo FGM/C [27]. Nell Williams (2014), wrote about Ethiopian girls and women about FGM/C, which stated “every mother wants to keep her children close, under her care, the high school is far away. If they send her, she might get abducted” [28].

On the other hand, children with the higher age (young people) are more influenced by their peers and exposed to multiple media like Facebook and the internet, to learn the ill effect of FGC. On top of this, education plays a great role, and powerful, in changing the attitude of young people (particularly of young girls). Moreover, the training at the school level and awareness at the community level enable them to identify the harmful effect of FGC. This study is supported by a study done in Burkina Faso which stated that education plays a better role for girls and women, to understand the negative health consequences associated with FGC [29]. Another study in Egypt stated that women living in the urban areas, having a higher level of education/literacy, and those who were working, were more likely to support the discontinuation of FGC [14]. Other studies also stated that literate, better educated and employed women are more likely to oppose FGC [30, 31]. These imply that there is a need to work on women’s education and empowerment to change their positive attitude towards the practice for the abandonment of FGC.

One of the major findings of this study is that the majority of the young study participants supported the abandonment of FGC (82%). This could be due to the result of different awareness-raising campaigns in the regions, and the provision of FGC education in schools as compared to the previous times. Furthermore, school based awareness which emphasized FGC health complications, may contributed to the young people to support the abandonment of the practice. This study is supported by the study done among school boys and girls in the Somali and Harari regions which stated that, FGC cause problem during delivery (63.4%), cause infection (55.3%), cause pain during sexual intercourse (64.3% ), cause bleeding (44.7%) [32], and the majority (86%) of the respondents, condemned the continuation of the practice (FGC). Another study also reported that FGC causes bleeding, shock, genital tissue swelling, fever, infections, and problems with urination and wound healing [33]. This implies that FGC awareness based on its health complications may have potential power to facilitate the abandonment of FGC. The abandonment of FGC requires the effort of the entire community, to create an enabling environment for change [34]. Therefore, efforts to end the practice need a collective approach to address the entire communities in ways that can decrease the social expectations to perform FGC [31]. To promote community-wide abandonment of FGC, community-led programs based on participatory methods, and women empowerment is curtailed [34]. The result of this study is in line with the study by UNICEF which stated that a moment of public affirmation of commitment is required to abandon the practice, so that each individual is assured that other community members are willing to end FGC, and leads Families able to change their social status of cutting and protect their daughters from harm [35]. Another study also supported the abandonment of FGC by stating that information gained from multiple sources might help the participant to internalize the problem and make an attitudinal change to design FGC abandonment [36]. To bring FGC abandonment to reality, the approaches should be based on holistic, community-based, and human rights education, to promote the social transformation among individuals, families, and to the entire community, who would forever end FGC, and quickly motivate the remainder of the intermarrying population [11, 37].

In contrast, the qualitative study done in the Somali and Harari regions/Ethiopia stated that women living in the Somali region were more likely to support the continuation of FGC. As expressed by a 52- year-old female from Somali, *“We Somali women know the harmful effect of FGC. We are suffering throughout our life. Despite this all, no Somali mother wants her daughter left unmarried due to this existing culture, “Marriageability” that is why we support the continuation of FGC.”* [20]*.* There are varies studies which support the continuation of FGC, such as the study done in Egypt, which stated that up to 82% of the women supported the continuation of FGC [30]. Studies done in Hargeisa district/Somalia also stated that although people are aware of the harmful effect of FGC, they support the continuation of the practice and there is a strong resistance towards the abandonment of the practice [35, 36]. Other studies in Ethiopia stated that families are forced to practice FGC, due to fear of stigma and discrimination by the community. The study further stated that the practice is extremely common among illiterate, rural resident, and Muslim religion, due to strongly and personally held beliefs for deviating from community norms, and fear of GOD’s punishment, if not cut their daughters [24, 31, 37, 38]. The aforementioned studies, however, targeted the attitudes toward the practice among adult people, while our study focused on young people, who are students, which implies a high level of literacy and potential exposure to social media among participants in our study.

The present study has limitations. The self-reported answers might have been to some extent in social desirability bias. Moreover, the study participants were students where 17.1% and 45.4% of females were literate in Somali and Harari regions respectively [39], while the majority of the people in the two regions are illiterate. Therefore, the generalization of the result of this study to the people in Somali and Harari regions should be avoided.

## Conclusions

The study result shows that multiple information channels that include school-based awareness campaigns were found to be the best way to support the abandonment of FGC. That means information from different sources is the powerful tool to disseminate the harmful effect of FGC among the study population. This was reflected in the study by the high proportion of support toward the abandonment of FGC, and the high number of Female study participants who didn’t experience circumcision. Although the study result demonstrates an increased perspective towards the abandonment of FGC among the study subjects, FGC abandonment is the unfinished agenda which is highly linked and reinforced by societal norms. Thus, to quicken the perpetuation of FGC in the stated Regional States, awareness creating campaigns that change the attitudes of youths towards the practice should be delivered through various sources. In this regard, school-based education, school mini-media, and social media could play significant roles in changing the pupils’ attitudes towards the practice. More importantly, using the co-curricular activities to uncover the danger of this harmful practice could make students change their attitudes towards it (FGC).

## Additional file

Additional file 1: Knowledge of the study population towards FGC in Somali and Harari regions. (PDF 131 kb)

## Abbreviations

AOR: Adjusted odds ratio
CI: Confedence Interval
FGC: FemaleGenital circumcision
FGM: Female genital mutilation
OR: Odds ratio
SPSS: Statistical package for social science
TV: Television
UNFPA: United Nations population fund
UNICEF: United Nation Children’s fund
WHO: World Health organization
