# Supplementary material for: Pupil’s perspectives on female genital cutting abandonment in Harari and Somali regions of Ethiopia
Source: BMC Womens Health. 2018 Oct 17;18:167. doi: 10.1186/s12905-018-0653-6 (PMC6192353; doi:10.1186/s12905-018-0653-6)
Supplement: Supplementary file 1 — Knowledge of the study population towards FGC in Somali and Harari regions. (PDF 131 kb) [file 12905_2018_653_MOESM1_ESM.pdf]

Additional file 1. Table not shown in the text

Knowledge of the study population towards FGC in Somali and Harari regions

Knowledge towards FGC	Total Frequency N=478	Percent (%)	Regions		Gender	
			Harari N=240	Somali N=238	Male	Female
Age of circumcision						
At birth	24	5	13(5.4%)	11(4.6%)	8(3.4%)	16(6.5%)
1-5 years	74	15.5	35(14.6%)	39(16.4%)	30(12.9%)	44(17.9%)
6-14 yeas	219	45.8	101(42.1%)	118(49.6%)	113(48.7%)	106(43.1%)
>14 Years	47	9.8	12(5.0%)	35(14.7%)	26(11.2%)	21(8.5%)
I don`t know	114	23.8	79(32.9%)	35(14.7%)	55(23.7%)	59(24.0%)
Type of FGC performed in the regions						
Sunna	132	27.6	26(10.8%)	106(44.5%))	73(31.5%)	59(24.0%)
Infibulations	128	26.8	42(17.5%)	86(36.1%)	52(22.4%)	76(30.9%)
I don`t know	218	45.6	172(71.7%)	46(19.3%)	107(46.1%)	111(45.1%)
The decision maker to perform FGC in the regions						
Mother	197	41.2	69(28.8%)	128(53.8%)	95(40.9%)	102(41.5%)
Father	25	5.2	10(4.2%)	15(6.3%)	9(3.9%)	16(6.5%)
Both	165	34.5	84(35.0%)	81(34.0%)	72(31.0%)	93(37.8%)
I don`t know	91	19	77(32.1%)	14(5.9%)	56(24.1%)	35(14.2%)
FGC performers						
Traditional practitioners	351	73.4	162(67.5%)	189(79.4%)	164(70.7%)	187(76.0%)
Own Mother	21	4.4	0(0%)	21(8.8%)	9(3.9%)	12(4.9%)
Health professionals	17	3.6	5(2.1%)	12(5.0%)	3(1.3%)	14(5.7%)
I don`t know	89	18.6	73(30.4%)	16(6.7%)	56(24.1%)	33(13.4%)

Support towards the abandonment of FGC among Somali and Harari regions, eastern Ethiopia

Support towards the abandonment of FGC	Total Frequency N=478	Percent (%)	Regions		Gender	
			Harari	Somali	Male	Female
Do you support the abandonment of FGC(n=478)						
Yes	391	81.8	194(80.8%)	197(82.8%)	195(84.1%)	196(79.7%)
No	87	18.2%	46(19.2%)	41(17.2%)	37(15.9%)	50(20.3%)
What is some of the efforts to support the abandonment of FGC (n=391)						
Community awareness	381	97.4	189(97.4%)	192(97.5%)	187(95.9%)	194(99.0%)
Punishment	10	2.6	5(2.6%)	5(2.5%)	8(4.1%)	2(1.0%)
The effort of the government (n=478)						
Awareness to the community about the ill effect of FGC	218	45.6	96(40.0%)	122(51.3%)	101(43.5%)	117(47.6%)
Took measure and punished the performers	112	23.4	72(30%)	40(16.8%)	54(23.3%)	58(23.6%)
I don`t know	148	31	72(30%)	76(31.9%)	77(32.2%)	71(28.9%)
The Effort of the school (n=478)						
Teaching in the classroom about the health risk of FGC	291	60.9	129(53.8%)	162(68.1%)	139(59.9%)	152(61.8%)
I don`t know	187	39.1	111(46.2%)	76(31.9%)	93(40.1%)	94(38.2%)
The effort of young people (n=478)						
Teaching the community about the harm of FGC	201	42.1	88(36.7%)	113(47.5%)	99(42.7%)	102(41.5%)
To expose the performers to the police	73	15.3	47(19.6%)	26(10.9%)	41(17.7%)	32(13.0%)
I don`t know what to do	204	42.7	105(43.8%)	99(41.6%)	92(39.7%)	112(45.5%)
The responsible person for abandoning FGC *(n=478)						
The government	246	51.8	103(43.1%)	143(60.6%)	130(56.5%)	116(47.3%)
The school	104	21.9	53(22.2%)	51(21.6%)	39(17.0%)	65(26.5%)
The region administration	126	26.5	60(25.1%)	66(28.0%)	42(18.3%)	84(34.3%)
The family	273	57.5	144(60.3%)	129(54.7%)	123(53.5%)	150(61.2%)

*All percentage in the responsible person for abandoning FGC is due to multiple responses
